# Annotations

**Published:** 1903-10-03

**Authors:** 


### Annotations.

### The Emotional Donor.

The money showered upon Arthur Batten, the man whose wife died of starvation in order to give what little food there was to her children, has drawn from Dr. W. Wynn Westcott, the coroner, some warning remarks about this kind of emotional charity. It is not a question of the case being a deserving and necessitous one, which is not disputed, but of the unreasoning generosity of people who are, if they but knew it, more anxious to soothe their own feelings than permanently to help their beneficiaries. There are many cases as tragic and deserving as those which draw the money out of people's pockets, which, however, lack the dramatic feature which appeals to an emotional public, or perhaps only lack the presence of a picturesque reporter with a sharp eye to copy. Dr. Westcott reckons that about four out of the eleven hundred inquests which he holds annually catch the public imagination, with the result that gifts are showered upon the few, while the many are left to their poverty and distress. Moreover, people who have been for a long time in the depths of poverty are often incapable of managing wisely a comparatively large sum of money. Without endorsing the opinion of Tennyson's "northern farmer" that "the poor in a lump is bad," it may be admitted that extreme poverty often shows a lack of the capacity of improving the position, even when the means of doing so are within reach. Dr. Westcott mentioned cases where a man who had been sober and industrious while he was miserably poor became a drunkard and a vagabond when these fairy gifts had relieved his necessities. To many of the poor the idea of working when one has money in hand is inconceivable. The present writer remembers a charwoman mentioning that some time before her husband had inherited £300. "And what did you do with it?" was asked. The woman looked up in surprise. "We spent it," she said simply. "We did no work until it was done." Similar conduct is by no means uncommon. Meanwhile societies which try to spend the money entrusted to them wisely are often in lack of funds, and the magistrates' and coroners' poor-boxes, from which suitable sums might be given by these gentlemen, who know all the circumstances of the various cases, are left nearly empty. But the public are slow to learn the lessons of true charity.

### The Alleged Increase of Insanity.

In a recent report presented by Mr. John Carswell as certifying physician in lunacy to the Glasgow Parish Council this question is very fully discussed. It is admitted that there is a steady increase in the number of those resident in asylums. But this, it is argued, is due not to an increased production of insanity but to a greater tendency on the part of the public to take advantage of asylums for the care of the elderly feeble-minded. Statistics are quoted to show that during the last three years there has been no increase in the number of those certified as insane between the ages of 15 and 45. It is only when the latter age is passed that the figures betray an upward movement. Further it is noted that the production rate at ages under 45 years keeps stable both in males and females, proving, it is suggested, that, whatever be the influences which determine the development of insanity, they operate equally on the two sexes. Mr. Carswell argues that, under modern industrial conditions, the real struggle for existence among the working and labouring classes only comes into operation in the middle and later periods of life. The younger and more active members of these classes can in large cities secure abundant employment, and their health is largely protected by public authority. Again, there is a growing movement in the direction of employing younger workers at the expense as it were of the middle-aged and elderly. Hence the latter, more especially if incompetent or of drunken habits, tend to succumb, and the collapse, in a certain proportion of instances, shows itself in the form of insanity. The changed attitude of public opinion in relation to rate-aided institutions and possibly an increased readiness under the pressure of modern competition to avoid the expense and charge of weak-minded relatives have led to an influx into the asylums of these elderly lunatics. It is in this way that the apparent increase of insanity is to be explained. From the same figures Mr. Carswell draws the comforting conclusion that the race is not degenerating. If such were the case evidences of hereditary defect would be expected to show themselves in the earlier and more active period of life. The statistics prove that this does not occur.

### A Testing Home for Reformed Drunkards.

The Church of England Temperance Society is doing a wise thing in establishing, as it proposes to do, a testing home for women who have been dismissed as cured from ordinary inebriate homes. Everyone knows cases which seem to be absolutely reformed, but which, when placed among the temptations of ordinary life, collapse immediately. The craving of a drunkard for drink cannot be tested in an inebriate home. After the first agony of craving the nerves and digestion gradually recover tone, and as there is no possibility of obtaining the accustomed stimulant the longing for it seems to abate. This seeming indifference to the old temptation is all the greater when, as in the best managed homes, the patients are kept occupied and interested all their waking hours. If the protected life of the home could go on for ever, it would be well for them. But this is impossible. In many cases the relations cannot go on for a long time paying the sums required for their maintenance in an institution; in all there are outside duties awaiting their return. But in many cases the return to the old environment and the old temptations means another fall. In the proposed testing home, which is to be situated in a village in East Anglia, the patients, passed on from an inebriate home, will be allowed a certain amount of liberty. They will be allowed to go out alone, and will have small sums of money in their possession. The amount of this money will be known, and they will be required to account for the way in which it has been spent. No doubt a considerable number of the patients will relapse, but for others this period of comparative liberty under kindly supervision will be the means of strengthening that self-control and will-power on which their ultimate reformation must depend.

